# The Herts and minds study: evaluating the effectiveness of mentalization-based treatment (MBT) as an intervention for children in foster care with emotional and/or behavioural problems: a phase II, feasibility, randomised controlled trial

**DOI:** 10.1186/s40814-017-0127-x

**Published:** 2017-02-22

**Authors:** Nick Midgley, Sarah Jane Besser, Helen Dye, Pasco Fearon, Tim Gale, Kiri Jefferies-Sewell, Karen Irvine, Joyce Robinson, Solange Wyatt, David Wellsted, Sally Wood

**Affiliations:** 1 The Child Attachment and Psychological Therapies Research Unit (ChAPTRe), The Anna Freud National Centre for Children and Families, 12 Maresfield Gardens, London, NW3 5SU UK; 2 Centre for Health Services and Clinical Research, The University of Hertfordshire, Health Research Building, College Lane, Hatfield, AL10 9AB UK; 3 Research Department of Clinical, Educational and Health Psychology, University College London, Gower Street, London, WC1E 6BT UK; 4 Research and Development, Hertfordshire Partnership University NHS Foundation Trust, The Colonnades, Beaconsfield Road, Hatfield, AL10 8YE UK; 5 Child and Adolescent Mental Health Services, Hertfordshire Partnership University NHS Foundation Trust, Forest Lane, Radlett, WD7 9HQ UK

**Keywords:** Looked after children, Mentalization-based treatment, Feasibility study, Clinical trial, Attachment, Reflective practice

## Abstract

**Background:**

A significant proportion of children in the social care system in England present with mental health problems, with the majority experiencing some form of emotional and behavioural difficulties. The most effective treatments for these children are currently unknown, partly due to a lack of robust, controlled studies. Researchers have identified a number of obstacles to conducting well-designed research with this population, making the need to test the feasibility of a randomised controlled trial especially important.

**Methods/design:**

This protocol outlines a two-arm, randomised control feasibility trial to explore the acceptability and credibility of mentalization-based treatment (MBT) as a treatment for reducing emotional and behavioural difficulties in looked after children and to test the possibility of addressing a number of methodological challenges to conducting high-quality research with this population. MBT is a relatively new intervention which, in the adaptation of the model tested here, includes many of the features of therapy identified in NICE guidelines as necessary to support children in care. The two arms are MBT and usual clinical care (UCC). The study will take place in Hertfordshire Partnership University NHS Foundation Trust with follow-up at 12 and 24 weeks.

**Discussion:**

This study will aim to ascertain whether it is worthwhile and feasible to progress to testing the intervention in a full-scale definitive randomised controlled trial (RCT). This study therefore has the potential to improve our understanding of the obstacles to conducting high-quality research with this very vulnerable population, and in the medium term, could help to improve the stability of foster placements and the emotional well-being of children in care.

**Trial registration:**

ISRCTN90349442

## Background

The term “looked after” was introduced in England and Wales by the Children’s Act, 1989 [1] and refers to children who are under 18 and have been provided with care and accommodation by a local authority’s (LA) children’s services. There were 69,540 children looked after (CLA) in England in March 2015, with the majority (61%) as a consequence of abuse, neglect and maltreatment [2]. Other reasons children become looked after include family dysfunction, child disability, parental illness or disability, and socially unacceptable behaviour. In a comprehensive survey conducted in 2002 [3], 45% of CLA aged 5–17 met the criteria for a psychiatric disorder, including conduct disorder (37%), and anxiety and depression (12%), but more recent studies have suggested that the figure could be as high as 72% [4]. This is in stark contrast to the figure for children with mental health conditions in the general population, which is estimated at 10% [3].

**Fig. 1:**
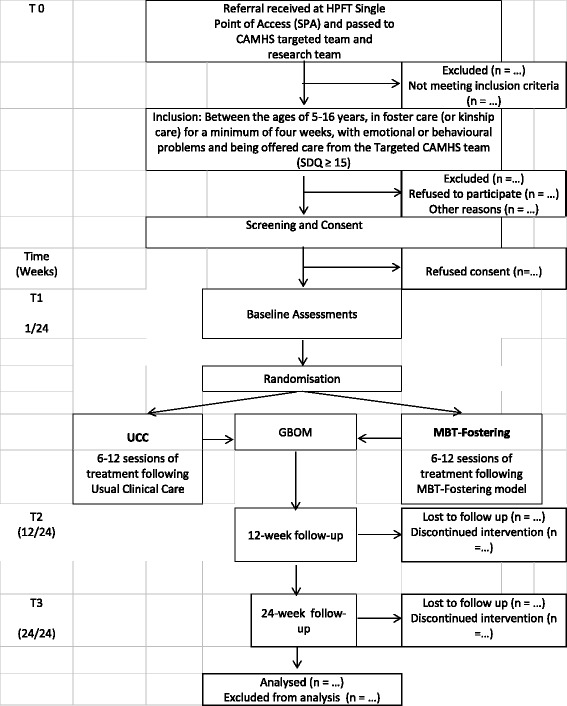
Study Flow Chart

CLA are therefore a vulnerable group with high levels of need and are at high risk of experiencing mental health problems [5, 6]. Children in care, when they present to mental health services, often do so with a complex presentation, including difficulties with emotional regulation and oppositionality, as well as challenges in forming and maintaining close social relationships, including with their carers [7]. Professional support systems are also frequently identified as lacking a shared understanding of the child’s needs or for a coherent approach to tackling common presenting concerns. As the mental health problems that can arise in these circumstances are significant predictors of future, long-term maladjustment [8–10], tackling the emotional and behavioural difficulties of CLA promptly and effectively is an important priority for health and social care professionals [11].

A recent analysis of USA data [12] found no difference in emotional or behavioural problems between CLA receiving a professional intervention relative to controls that did not. This analysis, in line with UK experience [13], provides little evidence that routine care has reliable positive effects on the outcomes for this vulnerable group, with concern that children may be offered a range of un-manualised and eclectic interventions for which evidence is lacking regarding their efficacy (13,14). Although some manualised approaches may be helpful for particular subgroups of children in care [14], the lack of a good evidence base for children with moderate emotional and behavioural problems referred to services means that those children are neither offered nor do they consistently receive high quality care. The 2013 National Institute of Health and Clinical Excellence (NICE) guidelines for CLA [11] concluded that “*the UK evidence base does not serve the needs of looked after children and young people as well as it might*” (p.86). It went on to argue that in most cases this is due to a lack of well-designed research investigating the effectiveness of mental health interventions in this population and drew attention to the *“lack of robust, adequately controlled, studies completed to a high standard*” (p.86).

Researchers have identified a number of specific obstacles to conducting high-quality research in a social care setting with CLA. These include the heterogeneity of this group of children itself, and the complexity of defining emotional health and well-being among them [15] ; the lack of appropriately designed and validated measures for use with this particular population [15] ; the lack of training in research methodology among social work professionals, creating a culture in which “practice-based wisdom” is valued over clinical trial evaluations [16]; and practical difficulties in accessing participants and gaining consent for participation of children in the care system [17].In a recent paper, [18], the authors report on the struggles they faced in recruiting CLA to a randomised trial, and describe how social care staff were often unable to prioritise work to recruit participants over their social care role, and expressed concerns that randomisation could be unethical or harmful to this vulnerable population of children.

The current study therefore focuses on the feasibility of conducting a clinical trial for children in foster care with emotional and behavioural difficulties, evaluating the effectiveness of an adaptation of Mentalization Based Treatment (MBT). MBT is a relatively new approach to psychological therapy, which grew out of developments in psychodynamic therapy and attachment research [19]. The approach draws especially on the finding that adults with early histories of neglect and attachment trauma often demonstrate a diminished capacity to mentalize, i.e. to make sense of the actions of oneself and others on the basis of intentional mental states, such as desires, feelings, and beliefs [19]. An evidence-base for MBT with adults with Borderline Personality Disorder has been developed [20, 21], and more recently studies have demonstrated the effectiveness of MBT for adolescents who self-harm [22], as well as for reducing violence in schools [23]. A range of adaptations of MBT have been developed for use with children and young people [24], including a family-based version, MBT-F; [25]. This approach has been used with a wide range of children including foster children, with promising results from one uncontrolled study [26].

The version of MBT tested in this study draws on the approaches set out above, but with a specific focus on the needs of CLA and their carers. The focus is on improving the core components of *secure attachment*, i.e. an emotional bond between the child and care giver in which the child has an expectation that their needs can be met [27], Although MBT has yet to be evaluated systematically in work with CLA, the approach has been manualised, drawing on the evidence-based principles of MBT for adults with borderline personality disorder, and includes many of the features set out in the NICE guidelines [11] as key elements of the best practice for work with CLA. Table 1 outlines the features of the MBT approach that converge with the NICE recommendations.

**Table 1:** The features of the MBT approach that converge with the NICE recommendations

Nice recommendation for looked after children and young people	Features of MBT relevant to the recommendations
Recommendation 6. Support professional collaboration on complex casework	▪ Promoting professional collaboration ▪ Ensuring that foster carers are included in the team around the child
Recommendation 7. Ensure everyone involved understands their role	▪ Promoting professional collaboration
Recommendation 8. Commission mental health services	▪ A focus on early intervention ▪ Promoting well-being and resilience
Recommendation 36. Train foster and residential carers	▪ Supporting social workers to have reflective conversations with foster carers that include emotional support and parenting guidance
Recommendation 37. Support foster carers and their families	“
Recommendation 38. Train supervisors	“
Recommendation 50. Develop a national core training module	▪ Reflective practice for those working with LAC
Recommendation 51. Train social workers to support looked after children and young people in education	“
Recommendation 52. Train independent reviewing officers to support looked after children and young people in education	“

The objective of this study is to establish whether it is feasible to conduct a full-scale clinical trial to evaluate the clinical and cost-effectiveness of MBT for looked after children who are experiencing emotional difficulties and their carers. This feasibility study will examine the challenges and establish procedures for a subsequent large-scale definitive RCT, including the recruitment of a control group that will receive usual clinical care (UCC).

As a feasibility study, the aims are to test the capacity to train mental health practitioners to an acceptable level of treatment integrity; to assess the feasibility of recruitment processes and study uptake; to establish acceptability and credibility of MBT as a treatment intervention for CLA; to establish the feasibility and acceptability to families of conducting a randomised clinical trial; to establish the feasibility of collecting resource-use data, for the purpose of calculating relative cost-effectiveness; and to constrain a preliminary estimate of likely treatment efficacy effect size (treatment outcome measures).

### Patient and public involvement

Patient and public involvement (PPI) has included feedback on the design of the study, instruments and assessments and the development of patient information and consent materials. They judged that the aims were relevant, the rationale was persuasive and there were no design aspects or ethical issues that should be improved or that they disagreed with. Feedback from service users, including a focus group with parents and interviews with children, was also drawn upon in the process of adapting the MBT model to the treatment of children in foster care, leading to an increased focus on treatment goals being set in relation to the needs of young people and on collaborative working.

## Methods/design

### Study design

The study is a two-arm, parallel group, single-centre feasibility randomised trial and will be conducted over a 24-month period.

### Setting

The study will be conducted in a child and adolescent mental health services (CAMHS) targeted service within a single NHS Trust: Hertfordshire Partnership University NHS Foundation Trust (HPFT). As of August 2014, there were 1023 children in care in the county. The CAMHS targeted team is a county-wide service which works collaboratively with children’s services. It provides a mental health service to CLA, as well as children and families who are actively working with social workers in children’s services.

### Participant selection

A total of 42 CLA and their carers will be recruited from routine referrals to CAMHS targeted team. The CLA team within HPFT is a cross-county service with an annual referral rate of 170 children per year. In 2013/14, 74.6% (*n* = 132) of children referred to the service would have met inclusion criteria for this study. Recruitment is planned for 12 months, suggesting that 32% of eligible children will need to be recruited to the study during the recruitment period. Regular monitoring of study progress will identify potential problems early and allow any corrective action to be taken.

### Inclusion criteria

The inclusion/exclusion criteria listed below were chosen based upon the eligibility requirements for children to receive a service from the CAMHS targeted team. The inclusion criteria are primary- and secondary-school age children (aged 5–16), in foster care (or kinship care) for a minimum of 4 weeks, referred to the CAMHS targeted team and accepted as an appropriate referral following the consultation meeting with the professional network, with emotional or behavioural problems (based on a score on the Strengths and Difficulties Questionnaire ≥15, which is Trust’s own criteria for young people to access the CAMHS targeted team).

### Exclusion criteria

Participants will be excluded if it is either an emergency/crisis referral, where an immediate response to a significant risk is required, or the referral is specifically for a psychiatric assessment in specialist CAMHS.

### Procedure

#### Interventions

All therapy offered by the targeted CAMHS team is up to 12 sessions. Clinicians employed by the CAMHS targeted team have varied training, including social work, clinical psychology, cognitive behavioural therapy, person centred therapy, supportive counselling, play therapy and family therapy. Decisions about which elements of usual clinical care to use for each child are made on the basis of a “Choice and Partnership Approach” (CAPA:[13]).

MBT is a short-term manualised treatment equal in length to UCC (i.e. up to 12 sessions) and delivered by existing CAMHS clinicians employed by HPFT. The MBT approach pays particular attention to promoting mentalizing in the foster carer and developing reflective practice for all professionals working with the referred child. The approach includes a combination of psycho-education about attachment and mentalizing in children with histories of maltreatment; consultations with the professional network around the child, when required; and direct relational work, tailored to the needs of each foster family, aimed at helping foster families understand their foster child’s needs and feelings, encouraging sensitive parenting and tackling problematic patterns of foster family interaction. MBT training of therapists will take the form of a 4-day group format including a 3-day intensive course, with a further 1-day follow-up session, provided by a systemic family therapist with expertise in using MBT with foster carers. Training will end with a videotaped assessment of performance (see https://spaces.xememex.com/tiddlymanuals/). Efforts will be made to keep “contamination” to a minimum by means of separate, fortnightly supervision arrangements for the two groups of clinicians. In order to randomly select the clinicians working in each arm of the study, whilst ensuring a balance in terms of clinical experience, the six staff will be paired by the service based on their length of time in role (experience in months/years), and subsequently ordered alphabetically by the study statistician who will generate a random sequence to randomise the suggested pairs of therapists into the MBT and UCC groups. If any member of staff has previous training in MBT, they will be automatically assigned to the MBT arm of the study.

### Participant identification and recruitment process

The study will build on the existing clinical pathways for referral to the CAMHS targeted team. As part of usual clinical practice, all referrals to CAMHS are received via the HPFT single point of access (SPA), and include a written referral, a copy of the Strengths and Difficulties Questionnaire (SDQ), and confirmation from the social worker that parent/s or legal guardian (henceforth referred to as “parents”), where appropriate, have given informed consent to the clinical referral. Please see Fig. 1 below for a flowchart of participant progression through the study, from referral to CAMHS to data analysis.

### Consent/assent process

In all cases, recruitment and consent will be the responsibility of the research fellow, clinical studies officer or trial manager (TM). The nature of the consent that will be required for the child to be recruited into the study will be determined by the child’s legal status and their age. Informed consent to take part in the study must be received from *at least one person holding parental responsibility for every child under 16 who meets the inclusion criteria for the study.* For children on *full or interim care orders*, this will be a representative of the local authority; for children on *voluntary placements*, this will be at least one of the child’s birth parents.

Whilst a child under the age of 16 is not considered to be competent in law to provide consent, many children over the age of 12 will be determined as Frazer competent and will therefore be able to provide consent as to whether they wish to be considered to take part in the study. Children will be provided with age-appropriate information sheets and given reasonable time to consider the study. Their assent will then be obtained if they are 12 or under, and written consent obtained for 13–16-year-olds. Where the child is on a full- or interim care order, a letter and information sheet will *additionally* be sent to parents, via their social worker, with details of the study and confirmation that their child will receive therapeutic care regardless of whether the child participates in the study. They will be informed that they may contact the study team if they have any objections to their child being part of the research via an “opt-out” method of consent. This method has been used and recommended previously [28].

As a feasibility study, data will be collected on how many participants do not speak sufficiently fluent English to complete the research measures, but they will not be excluded from the study. For those participants, although there may not be validated versions of the measures available in all languages, participants will be supported to complete the measures with the aid of an interpreter.

### Randomisation method and blinding

Individual participant randomisation will be utilised for the duration of the study with children assigned to one of the two treatment arms (MBT or UCC). Randomisation will be managed by the Clinical Trials Support Network (CTSN) at the University of Hertfordshire (UH), and will be requested and actioned electronically by the TM via the online secure data management system. Randomisation will be stratified by age (5–11 and 12–16), sex and whether the participant has siblings who are eligible, and otherwise randomly allocated. Randomisation will occur as soon as possible after consent has been obtained and the baseline assessment completed.

Six therapists are engaged in the study, and were placed into three pairs based on experience, ranging from senior to newly qualified social workers. The therapists were then randomly allocated to a treatment arm within each pair (one to each arm).

It is not possible to blind the therapists to treatment allocation, and for pragmatic reasons a decision has been made not to blind the child and foster family. Blinding of the child and foster family is judged not to be desirable (to avoid the perception of manipulation by the study team), and would be difficult to achieve, given that the MBT intervention involves active collaboration around the use of a mentalizing approach.

The members of the research team collecting and coding the research data and the study statistician will be the only team members blinded to treatment allocation; therefore, randomisation code breaking will not be necessary during the study.

### The outcomes of the study

The key outcomes of the study relate to the feasibility of conducting a large-scale RCT. The specific feasibility outcomes are as follows, with details of the instruments, scales, assessments and interviews to be utilised:

1. The capacity to train mental health practitioners (treatment integrity):
  The *MBT Adherence and Competence Scale* [29], rating both adherence and competence.
  Therapist feedback forms, to provide qualitative feedback about the MBT training and experience of its use in practice.
2. The feasibility of the recruitment processes:
  A *Recruitment Log*, including number of children referred, meeting inclusion/exclusion criteria, completing baseline assessment, randomised into the trial and reasons for non-eligibility or non-participation. This data will help to determine how long it would take to recruit participants into a definitive trial and the necessary referral levels/number of services that should be involved.
3. The acceptability and credibility of MBT as a treatment intervention for CLA:
  A *treatment attendance form*, recording non-attendance, and withdrawal with reasons. UCC group includes details about planned/received treatment.
  The *Experience of Service Questionnaire* (CHI-ESQ; [30]) in child, adolescent and carer formats, assessing service satisfaction.
  The *Experience of Therapy and the Research Process Interview* [31]; a qualitative interview with foster carer (and child, where appropriate) examining the experience and acceptability of the intervention.
4. The feasibility and acceptability to families of conducting a randomised clinical trial:
  The *Experience of Therapy and the Research Process Interview* [31] includes the family’s experience of, and barriers to participation in the trial. This qualitative data, along with data from the Case Report Form (CRF), will be used to assess feasibility and acceptability of the trial procedures themselves. This would include (a) the process of consent and randomisation and (b) the response rates for primary and secondary outcome measures, including retention at each of the three data collection time points and level(s) of missing data.
5. The feasibility of collecting resource-use data:
*Child and Adolescent Service Use Schedule* (CA-SUS: [32]; assessing resource use.
*Child Health Utility 9D* (CHU9D: [33]); assessing health-related quality of life.
6. To constrain a preliminary estimate of likely treatment efficacy effect size (treatment outcome measures), the following *treatment outcome measures* will be used to assess the effectiveness of the intervention in the definitive trial. For the feasibility RCT, assessment of the treatment outcome measures will be undertaken to support effect size estimation and to inform power estimation for the definitive trial. All the questionnaires will be used for the age ranges specified below, which indicate the age groups they are validated for.

Primary treatment outcome measure:

- *Strengths and Difficulties Questionnaire* (SDQ: [34], the routinely used clinical tool assessing emotional and behavioural difficulties in children aged 2–17 in the care system. Primary outcome for child behaviour and emotional well-being will be the carer-rated SDQ, but teacher-rated and young-person (11–17 years) rated versions will also be used, as appropriate.

Secondary treatment outcome measures:

- *Brief Assessment Checklist* (Child and Adolescent Versions—BAC-C [5–11] and BAC-A (12–16) [35] a caregiver-reported psychiatric rating scale, for children and young people, complementing the SDQ.
- *Revised Child Anxiety and Depression Scale* (RCADS: Child (8–16) Version and Parent Version: [36], a measure of anxiety and depression already used as part of routine outcome monitoring in CAMHS targeted team in Hertfordshire.
- The *Parent Stress Index*—*Short Form* (PSI-SF:[37]) used to assess carer wellbeing and the carer-child relationship.
- The *Parenting Efficacy Scale* [38], a measure of beliefs and confidence about parenting skills.
- The *Parenting Scale* [39], assessing parenting practices, including over-reactivity, a crucial focus of the study.
- A *Five*-*Minute Speech Sample* (FMSS, [40]), coded using the Reflective Functioning coding Manual [41] to assess the caregiver’s capacity for reflective functioning (mentalizing).
- *Goal-based Outcome Measure* (GBOM: [42]), assessing service user defined treatment outcomes.
- A *Significant Events* log recording significant life-events, including placement breakdown, involvement with youth justice system and school exclusions.

### Data collection

Data will be collected at baseline (T1), and then after 12 weeks (T2) and 24 weeks (T3). Table 2 below outlines the information collected at the different time points. The visit will take place at a time and place convenient to the foster carer, either at the foster carer’s home or at an HPFT site, and is estimated to take up to 2 h.

**Table 2:**
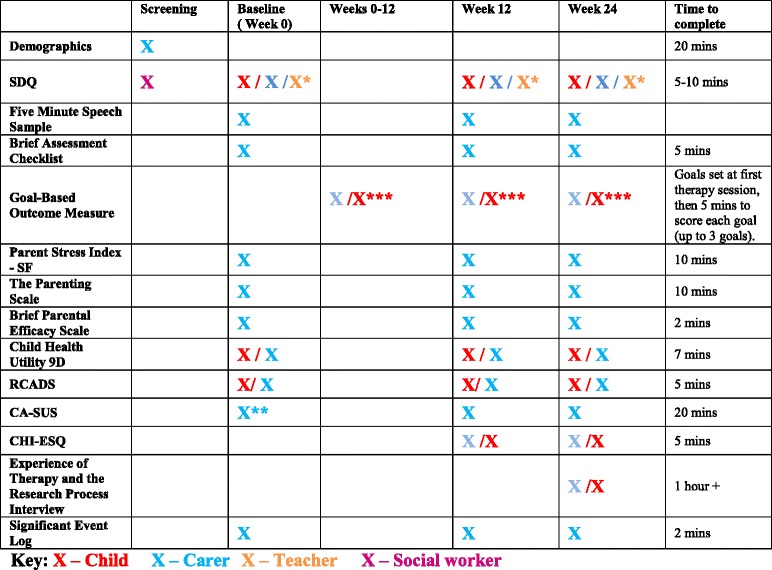
Schedule of Assessments

Table 2 shows the schedule of assessments. During the baseline assessment, after informed consent has been obtained, the participants will provide fuller information about their demographic status and history as outlined in the CRF. This basic data set will be followed by completion of the baseline outcome assessments.

### Treatment phase (0–12 weeks)

As part of routine practice, the therapist agrees with the child and the carer the goal(s) (usually up to three) for the therapy and goals are reviewed periodically throughout the therapy sessions. These goals are written down and recorded on a Goal-Based Outcome Measure (GBOM) Record Sheet.

The therapist will complete the Treatment Attendance Form over the period of therapy (0–12 weeks) for all participants, for all therapies (MBT and UCC). This includes details of the type of therapy utilised within the session (e.g. play therapy, CBT, and MBT). If the participant did not attend their scheduled therapy session, this will also be recorded with a reason, where possible.

In order to monitor adherence to the MBT model and differentiation from UCC, if the participant has consented to sessions being recorded, the therapist will audio-record each treatment session. At the end of the study, recordings for two of the families for each clinician in both arms of the study, selected randomly, will be reviewed by independent raters using an adaption of the MBT Competence and Adherence Scale to establish both treatment integrity and treatment differentiation.

### Follow up research visits

The T2 visit will occur at 12 weeks post-randomisation, which may or may not be after therapy has been completed. At 24 weeks from randomisation (T3), the final follow-up visit will take place. These assessments will consist of the outcome assessments completed at the baseline visit, plus the GBOM Scoring Sheet, the Experience of Service questionnaire, and record any significant events that may have occurred in the preceding weeks since the last assessments. In addition to the T2 measures, the T3 visit will also include a semi-structured interview in-person with the carer (and the child, where appropriate) about their experience of therapy and the research process. With consent, the interview will be audio-recorded. The interview will take place after all other assessment measures have been completed.

### Trial governance

The chief investigator (CI) will be accountable to the sponsor (the Anna Freud National Centre for Children and Families-AFNCCF) and will hold overall responsibility for the trial, including submission of required progress reports to the NIHR and NHS ethics committee, deliverables, financial statements and the correct use of funds.

The core study management team will consist of Dr Nick Midgley, Professor Pasco Fearon and Dr David Wellsted, who together will take responsibility for monitoring the overall progress of the study, including both data and budget management, and supervising the RF(s). The TM will manage the day-to-day running of the study with the support of the Clinical Trials Support Network.The CTSN will provide data-management and on-going data monitoring, including randomisation procedures, setup of the online database and secure management of confidential participant data. Co-investigators who will provide support to the study in relation to their specific areas of expertise. A study steering committee (SSC) has been set up to monitor progress and advise, meeting five times (0, 6, 12, 18, and 24 months), and will be responsible for monitoring patient safety.

### Planned analysis

The purpose of the statistical and qualitative analysis is to evaluate the feasibility of undertaking a full clinical trial. The strategy employed is therefore to describe aspects of the data following the six aims outlined above; the approach to each aim is described in turn.

### Aim 1: evaluation of treatment integrity

The primary data considered will for the first aim be the MBT Adherence and Competence Scale. The data for each MBT therapist (*N* = 3) will be tabulated to determine whether the therapists are consistently achieving at least an “adequate” rating on the target therapeutic qualities on the scale. Should any of the therapists not achieve the target set, the qualitative data will be evaluated to determine the extent to which the reasons could potentially compromise a training regimen for therapists. The data for each UCC therapist (*N* = 3) will be tabulated to determine whether there is a statistically significant difference between them and the MBT therapists in the level of mentalizing techniques used in sessions, i.e. to establish whether there is differentiation between the two treatment arms.

### Aim 2: feasibility of recruitment

The recruitment data will be presented in terms of absolute numbers and by proportions. In particular, the conversion of CLA referred to the CAMHS targeted team into children randomised to the study will be estimated as a key indicator of the feasibility of undertaking a full trial. The reasons for being excluded from the study at each point will be tabulated. The reasons for drop-out or exclusion will be examined for any consistent trends that may be addressed in the study design.

### Aim 3: acceptability of MBT as an intervention

For this aim, the primary data will be the extent to which children and carers attended therapeutic sessions as scheduled and the Experience of Service Questionnaire (CHI-ESQ-[30]). The analysis will tabulate therapeutic attendance, using both median and range data, and seek to determine particular trends where possible. It may be possible to evaluate the extent to which non-attendance is related to particular demographic or clinical factors crudely via regression models, or via stratification of the sample. Listing and classification of significant events during the children’s treatment may also help to highlight different effects on attendance patterns.

The acceptability of MBT as an intervention will also be assessed qualitatively, using the *Experience of Therapy and Research Process Interview.* A thematic analysis [43] will be conducted, to identify which elements of the intervention were experienced as acceptable or non-acceptable, and this will inform further development of the MBT intervention model if a full clinical trial is undertaken.

### Aim 4: feasibility and acceptability of randomisation & research procedures

The primary aim of this evaluation will be to examine data completion and retention across the study once the child has been randomised to a treatment arm. Data completion rates will be documented across all the outcome measures, and by treatment arm, and by time point. The analysis will seek to determine whether data completion falls below 75%, and potential differences between treatment arms, responding groups (children, families, teachers) and by subgroups (sex, age [≤11 or >11]), or any other demographic or clinical factor.

The study allows for a 30% drop out at the last follow-up [44]; and the data will be examined similarly for trends in the drop-out of children and their families. This will be tabulated according to treatment and subgroup as outlined above. Drop-out will be matched (tabulation) to the attendance data and to significant events to determine the extent to which drop out is determined by these factors. Regression models may be evaluated whether these factors are potentially informative and the study sample size allows.

The acceptability of randomisation, as well as other elements of the research design, will also be assessed qualitatively, using the *Experience of Therapy and Research Process Interview.* A thematic analysis [43] will be conducted to identify which elements of the research design were experienced as acceptable or non-acceptable. In addition to the process of randomisation, this will include the assessment burden and the appropriateness of each of the research measures (e.g. whether the parenting measures were felt to be appropriate for the experience of foster carers). This will inform the design if a full clinical trial is undertaken.

### Aim 5: resource use data

A similar approach to evaluating (non) completion of the resource use scales (CAS-US and CHU-9D) will be applied as for Aim 4. In addition, an evaluation of the meaningfulness of the data collected on the CA-SUS compared to the costs incurred by the children involved in the study will be undertaken. The extent to which the costs appropriately capture the financial challenge that the children present to the NHS and Social Care will be considered.

### Aim 6: likely effect size of the intervention

The preliminary effect size estimated for this study is based on an assumed minimum difference in the SDQ of 2 points given an sd = 7 typical of this sample of children. This gives a conservative estimate for an effect size of .29, and the sample size is estimated to enable a lower limit of 0 to be rejected. The analysis will seek to estimate the effect size from the observed data, with an estimated lower limit as suggested by Cocks and Torgerson [45].

### Evaluating the feasibility of a full scale trial

Principal determinants of whether a full scale trial can be undertaken will follow evaluation of each of the study aims as outlined.

Bringing together all the study data will allow trial feasibility to be considered, including the following:

- The potential to train therapists to deliver the intervention as envisaged and the costs involved.
- That the external validity of the study is maximised by ensuring that drop-out from the study is within acceptable limits (30%), or that changes can be convincingly made to the study procedures to allow for improved retention in the study.
- That the internal reliability of the study data is maintained. Data completion for the study measures, and completion of the therapeutic intervention (attendance) will be evaluated to ensure that the completion rate is good (>75%) and that there are no obvious biases in data completion by any particular subgroup. The qualitative evaluation will be considered in informing particular changes that can be made to maximise the study procedures to improve data completion.

The final report will summarise the data to support clear decision to proceed to a full trial, and to inform development of the study procedures to maximise the quality of the trial design.

### Sample size determination and power

The sample size of 42 is primarily calculated to test consistency with desired primary outcome effect size. Following Cocks and Torgerson [45], a sample size sufficient to reject a lower limit for the effect size of 0 is estimated (assuming one sided *α* = 0.05, 1− *β* = .80). Assuming sd = 7 in the SDQ is representative, with a lower limit = 2 for meaningful clinical change, the effect size = .29. For a feasibility trial, to reject an effect size of 0 a sample size of 32 (16 in each arm) is required, or 42 children (21 per arm) allowing for 30% drop out.

## Discussion

Children who have been taken into the care of the state are among the most vulnerable members of society, with far higher levels of emotional and behavioural difficulties than the general population, and with poor long-term physical and mental health outcomes. Yet in their review of the evidence for effective interventions for this group [15], the National Institute for Clinical and Health Excellence (NICE) identified few UK-based controlled trials that were “sufficiently robust or transparent to answer basic questions such as what interventions work best, how, for whom, and over what period, and what is good value for money” [15] (p.82). The lack of data on cost-effectiveness is a particular obstacle for policy makers and commissioners, for whom difficult decisions need to be made about resources and funding.

In the light of these challenges, the present study aims to test a number of important feasibility questions and tests a number of methods to try and overcome the barriers that have been identified by previous researchers. Regarding recruitment, the protocol has been designed to ensure that social care staff are not relied on to identify potential research participants, and careful attention has been paid to ensuring that consent can be obtained from the relevant parties (including parents, foster carers, the LA and the children themselves) in a way that is timely, appropriate and fully informed.

In addition, this study tests the feasibility of collecting service use data in a way that would help to evaluate the cost-effectiveness of the intervention as part of a full trial and combines the use of standardised treatment outcome measures such as the SDQ, which are already widely used with CLA, and measures such as the BAC, which have been specifically designed to assess the emotional well-being of CLA. Moreover, by combining qualitative and quantitative data, as recommended by NICE [15] (p.82), this study aims to identify some of the key barriers to conducting clinical trials with this population, as well as identify potential ways to address these barriers.

The wide-ranging challenges involved in the present research mean that a feasibility study is the most useful design. The overall research question aims to identify the most cost-effective treatment strategy for these children and incorporates a range of measures to identify the intervention with the greatest potential for positive impact on emotional health and well-being. By testing the feasibility of a trial to answer this research question, our study contributes information of major importance to the NHS. Potential challenges or difficulties to completing a larger-scale trial would allow us to modify and improve our research protocol, or if necessary to not proceed, if the findings of the feasibility study suggested that this intervention does not have a significant chance of improving outcomes for users of the NHS. Our hypothesis is that the outcomes will be better with MBT than UCC for equivalent cost. Given the current effect estimate, for roughly every eight families seen, at least one more family would benefit with MBT than with UCC, giving a saving to public services of up to £15,382 per family per year (minus the £2000 cost of treatment). These savings are of course in addition to the benefit of helping more families with the distress of relational difficulties and emotional and behavioural challenges.

The study could improve the stability of foster placements and the relationships between individuals within foster families, including a strengthened sense of security and belonging for CLA. This study therefore has the potential for direct impact on the day-to-day practice of health service staff, to bring savings to the NHS and local authorities, education and criminal justice sectors and benefits for some of the most vulnerable users of the NHS in England.

### Trial status

The current study status is that ethical approval was obtained in December 2015 and the study opened to recruitment in April 2016.

## Abbreviations

AFNCCF: Anna Freud National Centre for Children and Families
CAMHS: Child and adolescent mental health services
CBT: Cognitive behavioural therapy
CI: Chief investigator
CLA: Children looked after
CRF: Case report form
CRN: Clinical Research Network
CSO: Clinical studies officer
CTSN: Clinical trial support network
HPFT: Hertfordshire Partnership University NHS Foundation Trust
LA: Local authority
MBT: Mentalization-based treatment
NHS: National Health Service
NICE: National Institute for Health and Care Excellence
NIHR: National Institute for Health Research
PIRG: Public involvement research group
PPI: Patient and public involvement
RCT: Randomised controlled trial
RF: Research fellow
RfPB: Research for patient benefit
SPA: Single point of access
SSC: Study steering committee
UCC: Usual clinical care
UH: University of Hertfordshire
